# Yes, you can do global, cross-cultural behavioral science research using existing survey firms

**DOI:** 10.1073/pnas.2418102122

**Published:** 2025-03-06

**Authors:** Charles Crabtree

**Affiliations:** ^a^Department of Government, Dartmouth College, Hanover, NH 03755

**Fig. 1.**
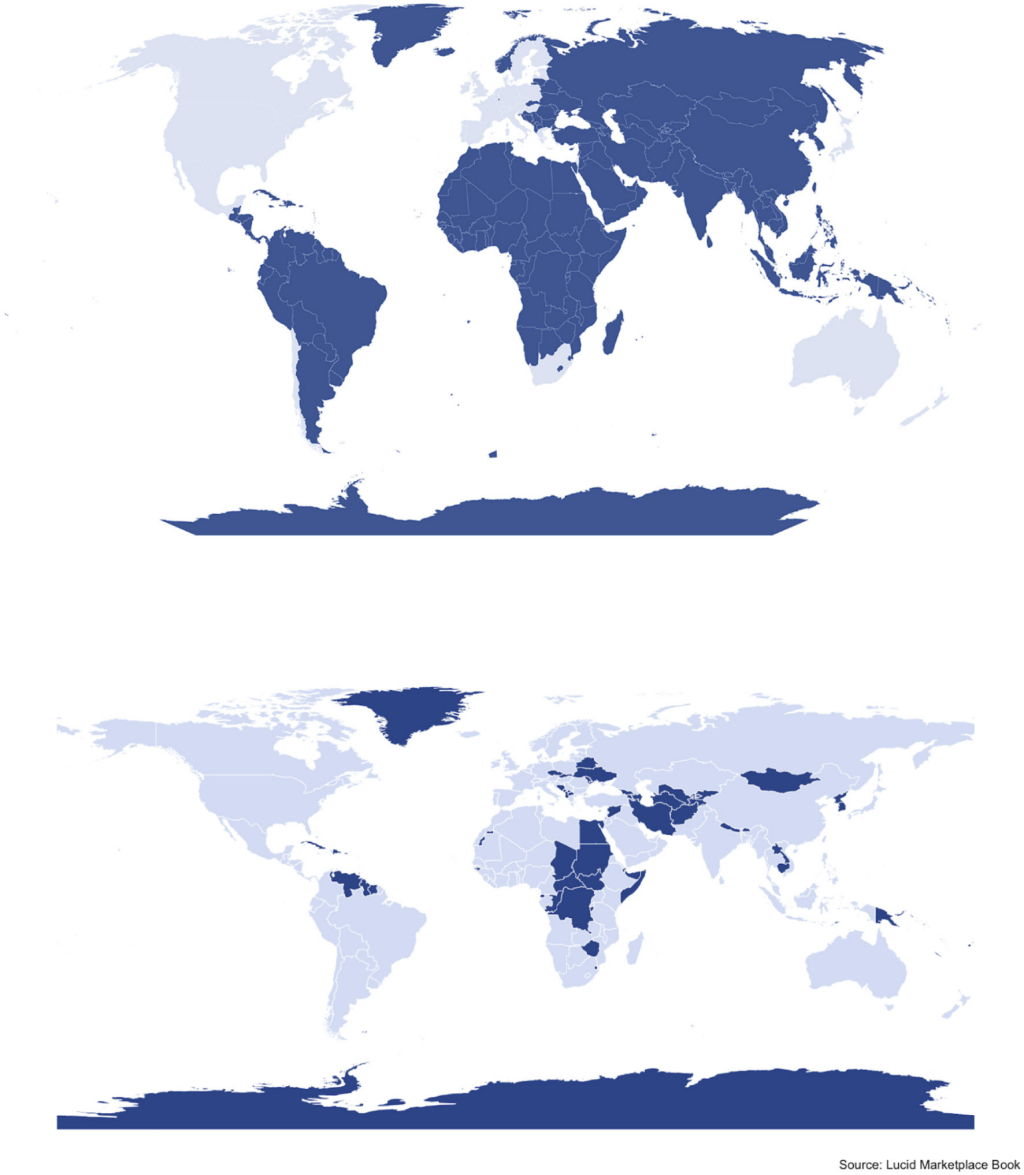
Countries not covered by Prolific (*Top*) and Lucid (*Bottom*) survey panels.

Gvirtz and Sabherwal (1) argue that global, cross-cultural research can be difficult because current research platforms—by which they seem to mean online survey sample providers or survey recruitment tools—lack sufficient geographic coverage. To support their argument, they point to the spatial limitations of two popular providers, Amazon Mechanical Turk (MTurk) and Prolific. They claim that MTurk has respondents in over 30 countries, but over 90% come from the United States (75%) or India (16%). Potentially more concerning, they state that Prolific does not cover the vast majority of countries. They illustrate this with a map of Prolific’s geographic boundaries, i.e., where the provider *does not* have more than 100 respondents registered (a low bar, as they admit). Their map, reproduced in the *Top* panel of Fig. 1, shows that Prolific only covers one country in each of the African, Asian, and South American continents. Based on their analysis of MTurk and Prolific, Gvirtz and Sabherwal (1) conclude that “to make progress, the academic community, including researchers and participant recruitment platforms, must be transparent about the inadequacy of the current system.”

The authors, though, base this conclusion on their experiences with two sample providers and strangely ignore other major online survey providers. They are missing one research platform, in particular—Lucid Marketplace (hereafter Lucid), a survey sample aggregator owned by Cint that helps researchers recruit subjects from many sources. Lucid has been widely used for many years and is often presented as providing higher-quality survey respondents than MTurk at a lower cost than Prolific. One indication of how established it is within the social sciences is that (2)’s subject validation study of Lucid has collected about 900 citations since it was published about 5 y ago.

Unlike MTurk and Prolific, Lucid offers extensive geographic coverage, and many researchers have used it to conduct cross-cultural studies (3). According to Lucid’s marketplace book, they have more than 44 million active users (i.e., those who logged in during the last month) from 110 countries (4). The *Bottom* panel in Fig. 1 shows Lucid’s geographic coverage using the color legend from ref. 1, i.e., dark blue countries have fewer than 100 respondents recently active on the provider. This map is dramatically different from the one that Gvirtz and Sabherwal show and leads to a substantially different conclusion about the ability of current research platforms to satisfy scholarly needs for geographically diverse subject pools: Researchers face a large range of geographic possibilities when determining case selection. Lucid is not the only provider that offers a broader geographic scope than Prolific–Dynata, another large and established provider, covers 46 countries, including four in Africa and twelve in Asia (5). While geographic and demographic coverage across current research platforms still needs to improve, scholars have the tools to make their research less WEIRD. Hopefully, they will use them, guided by theories about why the relationships they care about might vary across the impressive number of places where they can examine them.
